# Freeprotmap: waiting-free prediction method for protein distance map

**DOI:** 10.1186/s12859-024-05771-0

**Published:** 2024-05-04

**Authors:** Jiajian Huang, Jinpeng Li, Qinchang Chen, Xia Wang, Guangyong Chen, Jin Tang

**Affiliations:** 1 Zhejiang Lab, Zhejiang, China; 2 Dalian University of Technology, Liaoning, China; 3 The Chinese University of Hong Kong, Hong Kong, China

**Keywords:** Residue–residue distance prediction, Waiting-free, Feature representation

## Abstract

**Background:**

Protein residue–residue distance maps are used for remote homology detection, protein information estimation, and protein structure research. However, existing prediction approaches are time-consuming, and hundreds of millions of proteins are discovered each year, necessitating the development of a rapid and reliable prediction method for protein residue–residue distances. Moreover, because many proteins lack known homologous sequences, a waiting-free and alignment-free deep learning method is needed.

**Result:**

In this study, we propose a learning framework named FreeProtMap. In terms of protein representation processing, the proposed group pooling in FreeProtMap effectively mitigates issues arising from high-dimensional sparseness in protein representation. In terms of model structure, we have made several careful designs. Firstly, it is designed based on the locality of protein structures and triangular inequality distance constraints to improve prediction accuracy. Secondly, inference speed is improved by using additive attention and lightweight design. Besides, the generalization ability is improved by using bottlenecks and a neural network block named local microformer. As a result, FreeProtMap can predict protein residue–residue distances in tens of milliseconds and has higher precision than the best structure prediction method.

**Conclusion:**

Several groups of comparative experiments and ablation experiments verify the effectiveness of the designs. The results demonstrate that FreeProtMap significantly outperforms other state-of-the-art methods in accurate protein residue–residue distance prediction, which is beneficial for lots of protein research works. It is worth mentioning that we could scan all proteins discovered each year based on FreeProtMap to find structurally similar proteins in a short time because the fact that the structure similarity calculation method based on distance maps is much less time-consuming than algorithms based on 3D structures.

**Supplementary Information:**

The online version contains supplementary material available at 10.1186/s12859-024-05771-0.

## Introduction

The protein distance map is a two-dimensional matrix, where each value represents a residue–residue distance. Its binary form is known as a contact map. Lots of information can be directly obtained from them, such as secondary structure,^1^ motif,^2^ and interaction types^3^ concerning the kinds of residues. Besides, conserved patterns and structure motifs can be found [1] by analyzing the distance or contact map. Predicted distance or contact maps are widely used in remote homology protein detection [2–4], protein information estimate [5, 6], and protein structure research [7–9].

Distance maps or contact maps are utilized in remote homology protein discovery because it is rapid to predict and calculate the similarity between them. Although structure similarity measurement [10–13] and high-precision structure prediction [14, 15] are available, most existing structure alignment and structure prediction tools take substantial time and memory resources [16]. Considering the rapid growth of protein structure databases, a fast method to detect remote homology is needed. Therefore, a series of methods based on protein distance or contact maps have been proposed [2–4], which convert distance or contact maps to feature vectors and then use these vectors to calculate similarity.

Besides remote homologous protein detection, predicted distance or contact maps are widely used in protein information estimation, in which the potential structural information is captured by convolution calculations on the protein distance or contact maps, and the graph representation of proteins can also be obtained by using these maps. For example, Qiu et al. [5] integrate sequence, contact map, and GO label to predict protein functions. Chen et al. [6] use molecular docking simulation and graph representations of proteins based on contact maps discover two candidate drugs. Other common research work includes protein solubility prediction [17], key site prediction [18, 19], protein identification [20], and protein disorder region identification [21].

In the study of protein structure, residue–residue distance or contact maps are commonly used as collective variables to describe conformational changes in bio-molecular simulations. For example, Nassar et al. [7] employ residue–residue distances as biasing potentials in enhanced sampling MD simulations. Lubecka and Liwo [22] use residue–residue distances as restraints to improve structure simulations. Besides, the distance or contact maps are also used in protein design to assess the feasibility of producing a folded protein structure from a particular protein sequence [23]. Many protein domain segmentation methods also use contact information to segment protein domains [9, 24], which are based on the principle “as many intra-domain contacts as possible and as few inter-domain contacts as possible”. Distance or contact maps are also indispensable for many structure prediction algorithms. For example, Zheng et al. [25] fold non-homologous proteins by coupling contact maps with I-TASSER assembly simulations.

The widespread application of distance map prediction has attracted extensive attention from researchers. Barger et al. [26] and Rahman et al. [27] develop extended ResNets to predict distance maps; Si and Yan [28] hybridize 1D and 2D convolutions to increase the effective receptive field of the residual network. Madani et al. [29] develop an accurate protein predictor via hybrid generative adversarial neural networks. Rahman et al. [30] use three ResNets to predict the residue–residue distances within three ranges, and use the fourth ResNet to integrate their prediction results. Guo et al. [31] obtain multiple statistics from the multiple sequence alignments(MSAs) and then use them to construct four different feature sets for residue–residue distance prediction. Li et al. [32] train six ResNet models with the same architecture on various data subsets and ensemble them to make predictions. Deepdist [33] trains many models and ensembles them to predict real distance and distance boundaries at the same time, resulting in higher prediction accuracy.

However, most existing distance map prediction methods rely on MSAs but over half of all proteins are orphan proteins in standard sequence databases [34], and other related methods have their drawbacks. For example, most contact map^4^ prediction techniques [35] for orphan proteins have two limitations: (1) The information provided by contact maps is insufficient [36]; (2) Due to the employment of ensemble learning technology, most of them are time-consuming; yet, activities like remote homologous protein discovery are time-sensitive. Besides, although Alphafold-2 reliably predicts the protein structure with MSAs by using a variety of algorithms and engineering strategies such as the invariant point attention (IPA) module and recycling strategy, and then ESMFold and omegafold extended this prediction technology to orphan proteins by employing protein language models, their execution is time-consuming. To solve these challenges, we aim to develop a method to quickly and accurately predict distance maps for orphan proteins.

Firstly, we design a core model based on the properties of protein structure. Many local structures exist in protein, such as motif and domain, and the distance between any three residues must satisfy the triangle distance inequality constraint. Aiming to use locality and distance constraints, we design a model called R-former based on the triangular attention mechanism [14] and the proposed fast local microformer. Besides, considering the importance of the triangular attention mechanism in protein research, we also explain the triangle attention mechanism based on the residue–residue relationship, mathematical derivation, and feature representation.

Secondly, we propose group pooling to lower the dimensionality of protein representation, which will reduce the method’s computing cost. Transformer models trained with masked protein sequences depict the affinity of residues in a protein [37, 38] and frees us from relying on MSAs, but these representations are high-dimensional and sparse, which increases the computational cost of the prediction algorithm. We propose group pooling to solve this problem.

Thirdly, we provide error prediction and a new dataset to meet the needs of users. We design a deep learning framework named FreeProtMap based on R-former and group pooling to simultaneously output residue–residue distance and error predictions. We also compiled a dataset called dataset_4.05 by gathering recently published proteins(released on 2023.04.05) to help evaluate diverse prediction methods.

The contributions of this work are as follows:

- Aiming to improve the accuracy of predicted distance maps for orphan proteins, we propose an R-former that combines the triangular attention mechanism with our proposed fast local microformer. The R-former is designed to take into account both the locality and distance constraints in protein structures. Besides, a detailed explanation of the triangular attention mechanism is provided.
- Aiming to reduce computation cost, we propose group pooling to effectively reduce the dimensionality of protein deep representations.
- Aiming to meet the needs of users, we design a deep learning framework called FreeProtMap based on R-former and group pooling, which can simultaneously predict residue–residue distances and errors. We also construct a dataset using newly released proteins to evaluate the methods’ effectiveness.
- The proposed method predicts distance maps with a mean absolute error (*MAE*) of 2.32Å and a root mean squared error (*RMSE*) of 3.63Å on novel proteins. It predicts residue–residue distances more accurately than state-of-the-art structure prediction methods (ESMFold) with 3.74‰ inference time of it.

## Datasets and metric

### Datasets

We use the dataset curated by Yang et al. [39] as the base training dataset, which consists of 15051 protein sequences.

We adopt three test datasets provided by CASP14 and CASP15 competitions, and name them respectively as CASP_14D, CASP_14F, and CASP_15D datasets. The CASP_14D dataset includes all 15 protein domain fragments from the CASP14 competition. The CASP_14F dataset includes all 35 complete proteins from the CASP14 competition. The CASP_15D dataset includes all 44 protein domain fragments from the CASP15 competition. We could not find a complete proteins dataset in the CASP 15 competition, so we did not provide any related test results.

To evaluate the model’s performance in newly discovered proteins, we construct a new dataset by curating the recently released proteins (released on 2023.04.05) with PDB resolution below 2 Å. The dataset was designated 4.05_release dataset.

To reduce the impact of overfitting on model evaluation, homologous sequences are detected by using BLAST with an E-value cutoff of $1\times 10^{-3}$, which effectively filters out nearly all potential homologous sequences. We also further filtered out the redundancy with the training dataset and test datasets according to the 25% sequence identity threshold. Finally, there are 14618 proteins in the training set and 90 proteins in the 4.05_release dataset. We train the model with 11000 sequences and use the rest 3618 sequences as a validation dataset.

### Metric

Following the standard CASP definition [40], the distance between two residues is defined as the distance between their $C_b$ atoms ($C_{\alpha }$ for Gly). Many distance map prediction methods have focused only on residue pairs with real distances below 16 Å[33, 36]. However, recent research shows that accurately predicting the distances between residue pairs with real distances up to 36 Å is of great importance for determining the 3D structure [27]. We evaluate distance prediction on residue pairs with real distance within (0, 36 Å ).

The evaluation metrics consist of mean absolute error (*MAE*), root mean squared error (*RMSE*), R Squared ($R^2$) and mean deviation ($M^d$) for the above statistic. Their calculation methods are as follows:

1 $$\begin{aligned} MAE_{d}&= \frac{\sum _{i=1}^{n}\left| d_{i}-d_{i}^{p}\right| }{n} \end{aligned}$$

2 $$\begin{aligned} MAE_{p}&= \frac{\sum _{i=1}^{n}\left| AE_{i}-AE_{i}^{p}\right| }{n} \end{aligned}$$

3 $$\begin{aligned} RMSE&= \sqrt{\frac{\sum _{i=1}^{n} (d_{i}-d_{i}^{p})^2}{n}} \end{aligned}$$

4 $$\begin{aligned} R^2&= 1- \frac{\sum _{i=1}^{n}(d_i - d_i^p)^2}{\sum _{i=1}^{n}(d_i - \overline{d})^2} \end{aligned}$$

5 $$\begin{aligned} M^d_s&= \frac{\sum _{i=1}^{n}\left| s_i -\overline{s}\right| }{n} \end{aligned}$$

where $d_{i}$ denotes the real residue–residue distance, $d_{i}^{p}$ denotes the predicted residue–residue distance, *n* is the number of residue pairs with real distance below 36 Å, $AE_{i}$ denotes absolute error for the predicted residue–residue distance of the *i*th pair of residues, $AE_{i}^{p}$ represents the predicted absolute error about *i*th residue–residue distance, $\overline{d}$ denotes the average of real residue–residue distances, and *s* denotes the statistics such as *MAE*,*RMSE*, $R^2$.

## Methods

### Model architecture

**Fig. 1:**
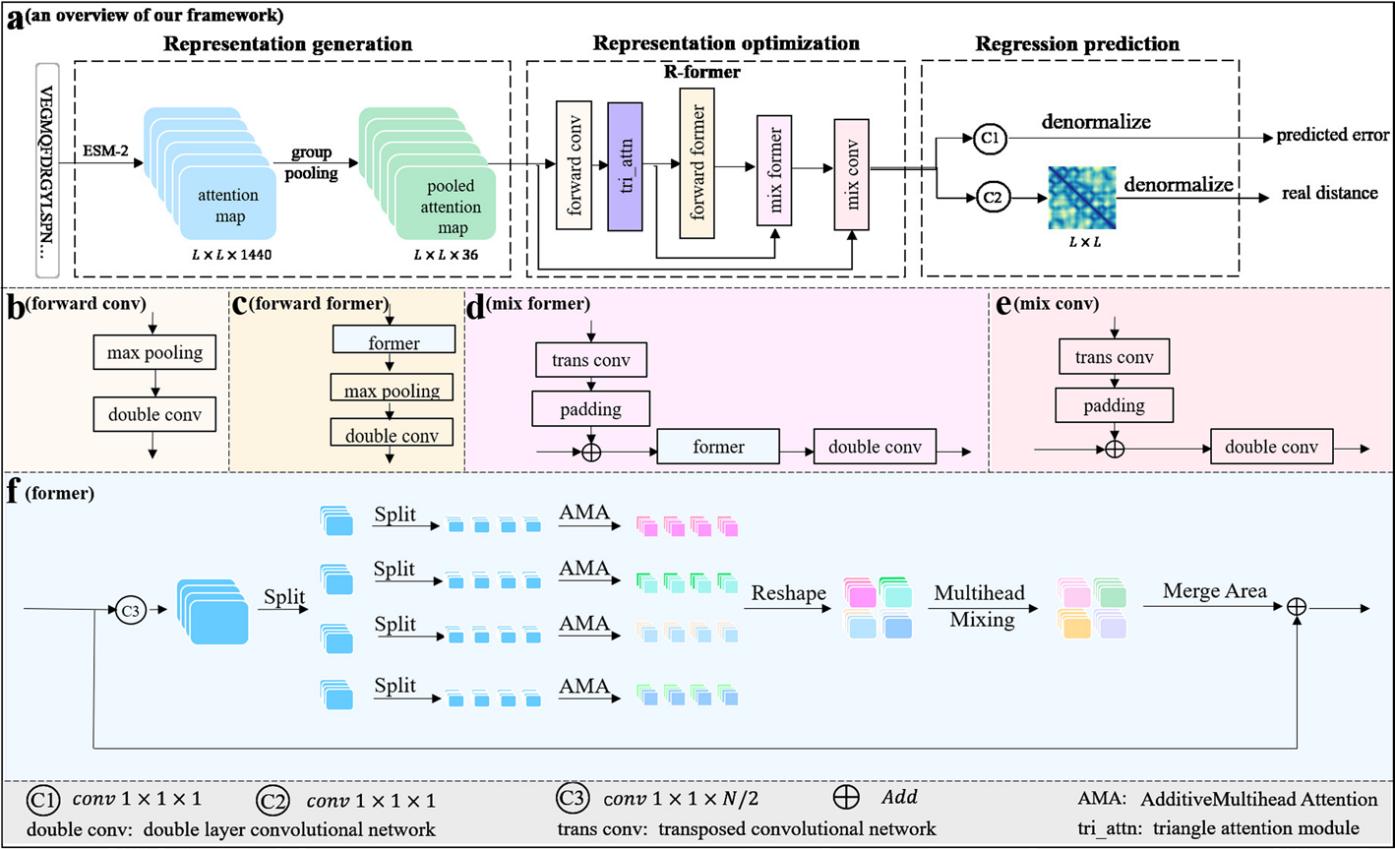
Illustration of our proposed framework, which consists of three modules: representation generation module, representation optimization module, and regression prediction module. In the representation generation module, ESM-2 generates attention maps of the input protein, which are then downscaled through group pooling to form the input representation (input feature maps). In the representation optimization module, the representation is further optimized by the R-former. The regression prediction module has two branches: one for distance map prediction and one for error prediction

The method described in this paper is illustrated in Fig. 1, which receives the protein sequence and outputs the predicted distance map, as well as the predicted absolute error on it. FreeProtMap carries out three processes sequentially: (1) Representation generation. (2) Representation optimization. (3) Regression prediction. More details are as follows:

#### Representation generation

**Fig. 2:**
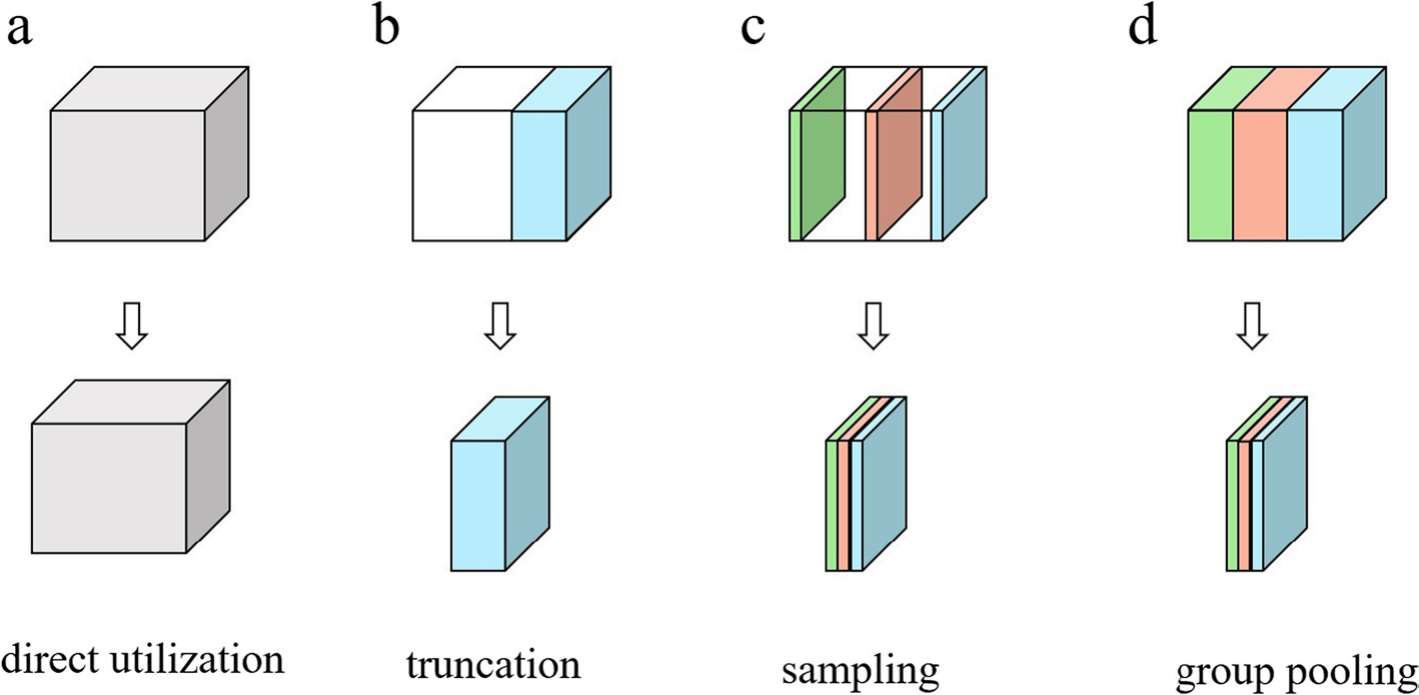
Common dimensionality reduction methods for attention maps of protein models. **a** Direct use: Make no processing on attention maps. **b** Truncation: Extract a portion of attention maps. **c** Sampling: Random sampling on attention maps. **d** Group pooling: Diminishing respectively the dimensions of attention maps in each feature subspace to maximize the retention of information

During representation generation, we implement the ESM-2 model to generate the primary feature representations and propose a novel group pooling method to extract the informative ones.

The ESM-2 produces high-dimensional sparse attention maps that indicate the relationship of each residue in the feature space [15]. High-dimensional sparsity refers to the phenomenon that a large number of zero values exist in feature maps or feature vectors. It leads to a serious computational cost, while simple dimension reduction methods result in significant information loss (Fig. 2). To solve this problem, we propose a group pooling method. It is founded upon the following principles:

1. The residue–residue relationship representations in attention maps contain much noise, therefore they will be separately dimensionality-reduced to mitigate the interference of these noises.
2. The residue–residue relationship representations potentially contained multiple types of residue–residue relationships. Each component of the representations may correspond to one kind of relationship. Therefore, the representations are divided into multiple sub-representations for processing.
3. Each above sub-representation is represented by its maximum response.

We represent the 3D tensor of attention maps as a set of 1D feature channel $X = \{X_i\}, i = 1\ldots N$, where $N = L \times L$ and *L* denotes the length of the protein sequence. $X_i = [X_i^1, X_i^2,\ldots ,X_i^j,\ldots ,X_i^M ]$, where $X_i^j$ is the component of $X_i$. $X_i^j(p)$ is the response at a specific channel position p over the set $\Omega$ of channel positions in the component. Therefore, the feature maps constructed by group pooling are given by:

6 $$\begin{aligned} f_i^j&= \max X_i^j(p), p \in \Omega \end{aligned}$$

7 $$\begin{aligned} f_i&= concat\left( f_i^1,f_i^2,\ldots ,f_i^j,\ldots ,f_i^M\right) \end{aligned}$$

8 $$\begin{aligned} f&= Assemble(f_i) \end{aligned}$$

For more specific details, please refer to Additional file 1: Appendix S1.

The group pooling method has tremendous potential for protein prediction applications. Currently, large-scale pre-trained models are widely used to acquire protein representations for various downstream tasks. However, these representations often suffer from high-dimensionality sparsity. While working on the input stage, exploiting the high-dimensional sparsity is unnecessary, and reducing effectively dimensionality can tremendously decrease computational expenses and alleviate model training challenges.

#### Representation optimization

The R-former proposed in this paper optimizes input representations for distance map prediction based on the two important properties of protein structure: locality and triangular inequality constraint.

*Modeling based on locality*. The locality is embodied as follows: (1) Many local structures exist in protein molecules, including $\alpha$-helix and $\beta$-sheet, as well as larger local structures like domain and motif. (2) In biological molecules such as proteins, the residue–residue distance changes in proteins molecules are continuous due to the presence of covalent bonds and non-covalent interactions. The distance between $x_i$ and $x_j$ is close to the distance between $x_{i \pm 1}$ and $x_{j \pm 1}$, where $x_i$ and $x_j$ denotes the *i*th and *j*th residue in the protein.

Besides, when designing a module based on the locality of protein structure, we need to consider the task properties: The patterns in the protein distance map are monotonous, so the module need a strong generalization ability. The local microformer [41] possesses three key features: significantly enhanced generalization ability, significantly enhanced local modeling ability, and lightweight. Therefore, we adopt the local microformer as the basic local modeling module. To fulfill the speed requirements for remote homologous detection, we have improved the attention calculation of the local microformer by implementing additive calculation [42]. These modifications result in the construction of a high-speed local microformer. We name it as a former module, which is illustrated in Fig. 1f.

The former module primarily performs the following four tasks (Fig. 1f):

(1) The input feature map is divided into several area blocks and each block is reshaped into one-dimensional sequences. Each sequence is denoted by $S_{i}$.

(2) These sequences are then sent into the multiheaded self-attention module. In this module, scaled multihead dot product attention is utilized to catch dependencies. The calculation method is as follows:

(2.1) Query, key, and value vector are retrieved by using three mapping modules $W^Q$, $W^K$, and $W^V$:

9 $$\begin{aligned} q_{i}=S_{i}W^Q; k_{i}=S_{i}W^K; v_{i}=S_{i}W^V \end{aligned}$$

(2.2) Query, key, and value vector are divided into H groups:

10 $$\begin{aligned} & q_{i}^{0}, q_{i}^{1},\ldots ,q_{i}^{H-1}=split(q_{i}) \end{aligned}$$

11 $$\begin{aligned} & k_{i}^{0},k_{i}^{1},\ldots ,k_{i}^{H-1}=split(k_{i}) \end{aligned}$$

12 $$\begin{aligned} & v_{i}^{0},v_{i}^{1},\ldots ,v_{i}^{H-1}=split(v_{i}) \end{aligned}$$

(2.3) Perform the following operations on $q_{i}^{m}$, $k_{i}^{m}$ and $v_{i}^{m}$.

(2.3.1) The query vectors are summarized into a global query vector by using additive attention:

13 $$\begin{aligned} \alpha _ {i} = \frac{exp(w_ {q}^ {T}q_{i}^{m}/\sqrt{d^h})}{\sum _ {i=1}^ {n}exp(w_ {q}^ {T}q_{j}^{m}/\sqrt{d^h})} \end{aligned}$$

where $w_q \in R^d$ is a learnable parameter vector and $d^h$ denotes the dimension of the $q_{j}^{m}$.

(2.3.2) The global attention query vector is computed as follows:

14 $$\begin{aligned} q^{m}= \sum _ {i=1}^ {N} \alpha _ {i} q_ {i}^{m} \end{aligned}$$

(2.3.3) The correlation of each pixel is calculated through the query vector and key vector within the group and the results are normalized:

15 $$\begin{aligned} p_{i}^{m} = q^{m}*k_{i}^{m}/\sqrt{d^h} \end{aligned}$$

(2.3.4) The additive attention weight of its *i*th key vector is computed as follows:

16 $$\begin{aligned} \beta _ {i} = \frac{exp(w_ {k}^ {1}p_{i}^{m}/\sqrt{d^h}}{\sum _ {i=1}^ {l-1}exp(w^ {T}_ {k}p_ {j}^{m}/\sqrt{d^h}} \end{aligned}$$

where $w_k \in R^d$ is a learnable parameter vector.

(2.3.5) The global key vector $k \in R^d$ is further computed as follows:

17 $$\begin{aligned} k^{m}= \sum _ {i=1}^ {N} \beta _ {i} p_ {i}^{m} \end{aligned}$$

(2.3.6) The weighted matching is performed on the value vector:

18 $$\begin{aligned} u_ {i}^{m} = k^{m} * v_ {i}^{m}. \end{aligned}$$

(2.4) The outputs of the multihead attention module are rearranged as follows:

19 $$\begin{aligned} y_{i}=concat \left[ u_{i}^{0},\ldots , u_{i}^{H-1}\right] \end{aligned}$$

(2.5) Transposed convolution is utilized to aggregate the different heads’ attention results contained in the outputs of the multihead attention module. Then the area blocks are merged into a feature map, and finally the feature map is blended with the original feature map by using a residual connection.

*Modeling based on distance constraints*. The distances between any three residues must satisfy the triangle inequality [14]. We reveal the principles of the triangular attention module in Alphafold-2 [14], which indicates this module can effectively introduce triangular inequality constraints in information modeling. The triangular attention module is illustrated in Additional file 1: Appendix S2. We believe that the triangular attention module works based on the following principle:

1. The residue–residue relationship and the residue–residue distance are closely related, as residues closer in space tend to undergo mutation together to achieve new stable states in which their physical and chemical states are coordinated. Besides, the attention maps that represent residual-residue relationships exhibit specific local patterns after processing, indicating a close correlation between residual-residue relationship representation and residual-residue distance.
2. According to the mathematical interpretation of vector dot product, $q_{(i,j)}k_{(i,k)}$ in the triangular attention mechanism can be transformed into $|q_{(i,j)} ||k_{(i,k)} |\cos \theta _{i}$.
3. Since the feature vector in the triangular attention module represents the residue–residue relationship and it is closed to distance relationship, $|q_{(i,j)} ||k_{(i,k)} |\cos \theta _{i} + b_{(j,k)}$ can be approximated as
  20 $$\begin{aligned} dist_{(i,j)} \times dist_{(i,k)} \times \cos \theta _{i} + dist_{(j,k)} \end{aligned}$$
4. According to the cosine law, Eq. 20 can be transformed into
  21 $$\begin{aligned} \frac{{dist_{(i,j)}}^2+{dist_{(i,k)}}^2-{dist_{(j,k)}}^2}{2}+dist_{(j,k)} \end{aligned}$$
5. Sum up,
  22 $$\begin{aligned}&softmax(q_{(i,j)} k_{(i,k)}+b_{(j,k)}) \\&\quad \approx softmax \left( \frac{{dist_{(i,j)}}^2+{dist_{(i,k)}}^2-{dist_{(j,k)}}^2}{2}+dist_{(j,k)} \right) \end{aligned}$$

The above analysis shows that in a triangle with three residues as vertices, when modeling the distance relationship between residues: If the sum of the two sides is much less than the third side, the resulting attention weight will be very low after applying softmax, which suppresses the propagation of incorrect distance relationships. This module only uses the residual-residual distance relationship, which satisfies the triangle distance constraint, to update other residue–residue distance relationships. Therefore, this module successfully introduces the triangle inequality constraint when modeling the distance relationship.

**Fig. 3:**
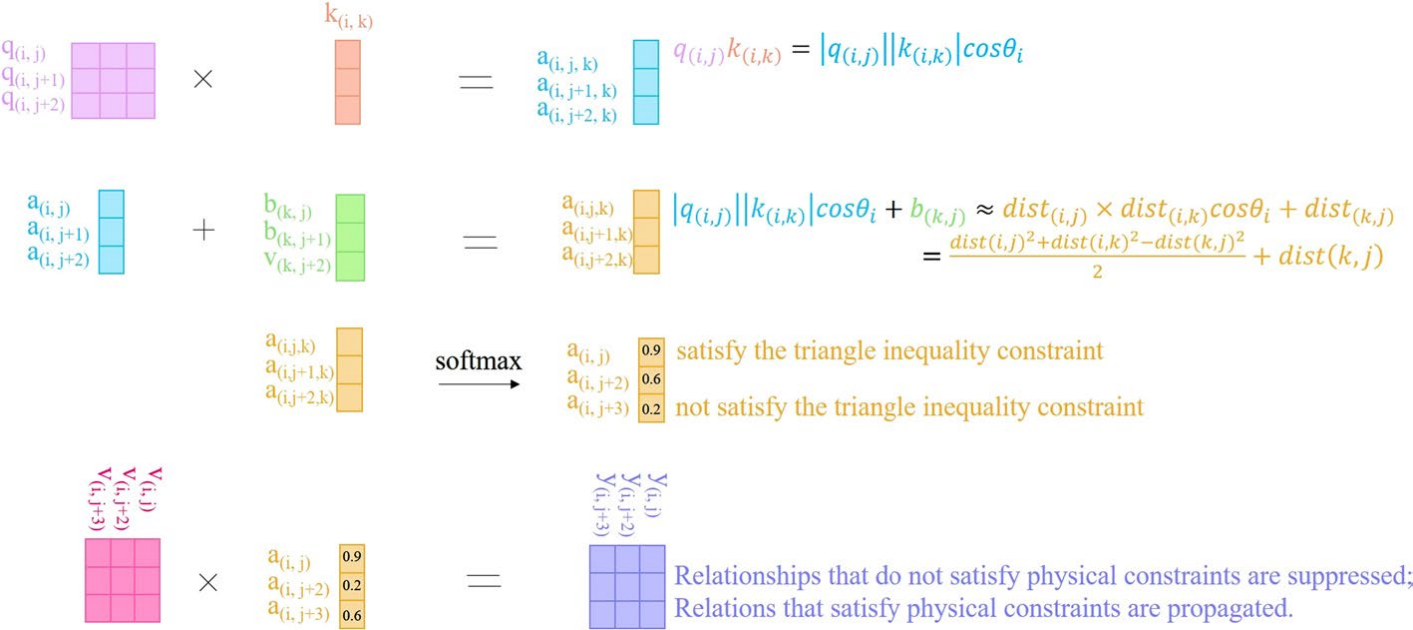
Illustration of the triangular attention mechanism. In the triangular attention mechanism, the feature vector represents the residue–residue relationship, which is mostly a distance relationship. Based on the mathematical definitions of vector dot product and cosine theorem, it can be understood how this module incorporates the constraints of triangle distance inequality into the modeling

To further clarify these principles, we offer Fig. 3 to show them.

*Design of R-former*. Based on these two modules, we carefully designed a hybrid structure called R-formers, which combines transformer and Convolutional Neural Networks (CNNs). The basic architecture of R-formers consists of a bottleneck structure and residual structures. The bottleneck structure aims to acquire a robust and high-dimensional representation of inputs and minimizing the risk of overfitting [43, 44]. The residual structure is designed to make the model easier to learn [45], as the neural network block only needs to learn a small residual. The triangular attention module is set on the second layer of the R-former to balance computational costs and prediction accuracy. The remaining hyperparameters for R-formers are chosen based on personal experience.

The R-former performs the following processes (Fig. 1a):

23 $$\begin{aligned} x_1&= conv^{f}(x_0) \end{aligned}$$

24 $$\begin{aligned} x_2&= tri\_attn(x_1) \end{aligned}$$

25 $$\begin{aligned} x_3&= former^{f}(x_2) \end{aligned}$$

26 $$\begin{aligned} x_4&= former^{m}(x_3,x_2) \end{aligned}$$

27 $$\begin{aligned} x_5&= conv^{m}(x_4,x_0) \end{aligned}$$

where $conv^{f}$ denotes the forward CNN (forward conv), $tri\_attn$ denotes the triangular attention module, $former^{f}$ denotes the forward former, $former^{m}$ denotes the mix former, $conv^{m}$ denotes the mix CNN (mix conv), and $x_0$ denotes the input representation generated in the first stage.

The $conv^{f}$ consists of a max pooling layer and a double layer CNN (Fig. 1b). The $conv^{m}$ consists of a transposed CNN, a padding operation, a fusion operation (Add), and a double-layer CNN (Fig. 1e). The $former^{f}$ and $former^{m}$ add the former module on the basic of $conv^{f}$ and $conv^{m}$ (Fig. 1c, d), respectively. The window sizes of all max pooling layers in Fig. 1 are 2. The filter number of convolutions are {64,64}, {128, 128},{64,64},{36,36} in double layer CNN of $conv^{f}$, $former^{f}$, $former^{m}$ and $conv^{m}$, respectively. Their sizes are all $3\times 3$. The filter number and size of transposed convolution in $former^{m}$ and $conv^{m}$ are 1 and $2\times 2$, respectively.

#### Regression prediction

We perform regression predictions for the distance map and error. This prediction process consists of two stages. Initially, the regression prediction layers output the predicted values related to residue–residue distance and absolute error (AE), which vary from 0 to 1. The regression prediction layers consist of a single-layer CNN with a kernel size of 1. In the second stage, the predicted values are denormalized to obtain predicted residue–residue distances and predicted absolute error. Specifically, the predicted values in the first stage are magnified N times to be transformed into the actual value. Considering that the real residue–residue distances range between 0 and 100, we set N to 100.

### Loss function

Our training strategy consists of two stages: First, we train the R-former and distance map prediction branch, and then we fix the R-former and distance map prediction branch before training the error prediction branch. We adopt a small loss strategy, calculating loss exclusively for residue pairings with actual distances under 36Å.

As MAE is more robust to outliers, it is the preferred loss function for our tasks with a wide numerical range and potential outliers, which is the mean of absolute differences between the predicted and real values:

28 $$\begin{aligned} \mathcal {L}=\frac{\sum _{i=1}^{n}\left| y_{i}-y_{i}^{p}\right| }{n} \end{aligned}$$

where $y_{i}$ denotes the true residue–residue distance or absolute error, $y_{i}^{p}$ denotes the predicted residue–residue distance or absolute error, and *n* is the number of residue pairs with actual distances under 36 Å.

### Implementation

We use the Adam optimizer with a weight decay of 0.01 to optimize the parameters for 30 epochs in the first stage and 5 epochs in the second stage. The initial learning rate is set to $1e^{-3}$. The batch size is 1. Our method is implemented on the PyTorch platform and trained with one Nvidia-A100 GPU.

## Results and discussions

We report the performance of FreeProtMap on distance map prediction tasks, along with the analysis of the model and results. Additionally, we report its performance on contact map prediction tasks.

### Comparison with other methods

#### Comparison with other methods on prediction accuracy

Due to the unavailability of MSAs-free distance prediction methods, we conduct a comparative analysis of FreeProtMap and state-of-the-art structure prediction methods, in which the predicted 3D structures are applied to generate the distance maps. Table 1 summarizes the experimental results with italic and bold highlighting the best results and the second-best results, respectively.

The results in Table 1 show that FreeProtMap greatly outperforms the best-published method on the test datasets, which verifies that our method is quite successful. More specifically, the proposed method achieves 2.32 Å in $MAE_{d}$, 3.63 Å in RMSE, and 0.88 in $R^2$ on the 4.05_release dataset, which outperforms the best-published method by 0.25 Å in $MAE_{d}$, 1.77 Å in RMSE, 0.21 in $R^2$. Furthermore, the mean deviation of each statistic shows that FreeProtMap exhibits relatively stable performance on new proteins. More specifically, the proposed method achieves 0.55 Å in $M^d_{MAE}$, 0.87 Å in $M^d_{RMSE}$ and 0.06 in $M^d_{R^2}$ on the 4.05_release dataset, which outperforms the best-published method by 1.69 Å in $M^d_{MAE}$, 2.63 Å in $M^d_{RMSE}$, and 0.14 in $M^d_{R^2}$. Besides, FreeProtMap can predict errors with an MAE of 2.45 Å.

We also evaluate the proposed FreeProtMap and compared methods on the CASP_15D dataset. Table 1 shows that FreeProtMap significantly outperforms the best-published method on this test dataset. More specifically, the proposed method achieves 2.50 Å in $MAE_{d}$, 3.83 Å in RMSE, 1.26 Å in $M^d_{MAE}$, 2.11 Å in $M^d_{RMSE}$ and 0.18 in $M^d_{R^2}$ on the CASP_15D dataset, which outperforms the best-published method by 0.61 Å in $MAE_{d}$, 1.10 Å in RMSE, 0.88 Å in $M^d_{MAE}$, 1.50 Å in $M^d_{RMSE}$ and 0.03 in $M^d_{R^2}$.

The exceptional performance of FreeProtMap can be attributed to five primary factors:

1. The attention maps after group pooling better characterize the information on protein residues.
2. The distance map has evident local patterns, and the former module is applied to enhance the local information modeling.
3. FreeProtMap benefits from the AlphaFold-2’s triangle attention module, which exploits the triangular constraint in distance maps.
4. The bottleneck structure of the R-former helps reduce overfitting [43, 44]. R-former faces a significantly lighter overparameterization than ESMFold. These two changes increase FreeProtMap’s performance on novel proteins.
5. Small loss strategies are employed during training to mitigate the influence of problematic data.

The proposed method does not achieve perfect accuracy maybe because there is a deviation in the experimentally measured PDB for the following reasons: (1) The static structures are determined under non-physiological conditions; (2) Different crystallization situations, different structure analysis technologies (NMR, X-ray, cryo-EM, etc.) and even different structure computation methods may lead to structure variation.

**Table 1:** Comparison of proposed approach with state-of-the-art methods for residue–residue distance prediction

Method	Source	The 4.05_release dataset						
		$MAE_{d}$	$M^d_{MAE}$	*RMSE*	$M^d_{RMSE}$	$R^2$	$M^d_{R^2}$	$MAE_{p}$
OmegaFold [46]	bioRxiv’22	7.05	7.27	10.92	9.80	0.49	0.47	–
HelixFold-single [47]	bioRxiv’22	7.56	2.66	11.00	**3.50**	0.38	**0.20**	–
RGN2 [48]	Nat. Biotechnol.’22	5.36	4.43	8.44	5.72	0.52	0.34	–
trRosettaX-single [49]	Nat Comput Sci’22	3.39	2.29	7.79	6.32	0.60	0.33	–
ESMFold [15]	Science’23	**2.57**	**2.24**	**5.40**	4.63	**0.67**	0.37	–
FreeProtMap	Ours	*2.32*	*0.55*	*3.63*	*0.87*	*0.88*	*0.06*	2.51

Method	Source	CASP_15D						
		$MAE_{d}$	$M^d_{MAE}$	*RMSE*	$M^d_{RMSE}$	$R^2$	$M^d_{R^2}$	$MAE_{p}$
OmegaFold [46]	bioRxiv’22	4.24	2.91	6.61	4.39	0.69	0.32	–
HelixFold-single [47]	bioRxiv’22	5.55	4.41	8.10	6.85	0.26	0.72	–
RGN2 [48]	Nat. Biotechnol.’22	4.95	2.53	6.21	4.64	0.73	0.28	–
trRosettaX-single [49]	Nat Comput Sci’22	3.78	2.22	5.32	4.16	0.78	0.27	–
ESMFold [15]	Science’23	**3.11**	**2.14**	**4.93**	**3.61**	*0.80*	**0.21**	–
FreeProtMap	Ours	*2.50*	*1.26*	*3.83*	*2.11*	*0.80*	*0.18*	2.63

Method	Source	CASP_14F						
		$MAE_{d}$	$M^d_{MAE}$	*RMSE*	$M^d_{RMSE}$	$R^2$	$M^d_{R^2}$	$MAE_{p}$
OmegaFold [46]	bioRxiv’22	*3.39*	2.58	**5.10**	3.93	*0.76*	*0.23*	-
HelixFold-single [47]	bioRxiv’22	4.80	7.16	7.16	4.91	0.38	0.54	–
RGN2 [48]	Nat. Biotechnol.’22	4.55	2.84	5.14	4.00	0.64	**0.27**	–
trRosettaX-single [49]	Nat Comput Sci’22	3.61	3.15	5.49	3.96	0.62	0.42	–
ESMFold [15]	Science’23	**3.52**	**2.54**	5.14	**3.89**	0.67	0.33	–
FreeProtMap	ours	3.54	*2.19*	*4.95*	*3.31*	**0.68**	0.28	3.61

Method	Source	CASP_14D						
		$MAE_{d}$	$M^d_{MAE}$	*RMSE*	$M^d_{RMSE}$	$R^2$	$M^d_{R^2}$	$MAE_{p}$
OmegaFold [46]	bioRxiv’22	2.43	2.04	3.91	3.27	0.84	0.18	–
HelixFold-single [47]	bioRxiv’22	6.07	4.79	9.23	7.62	0.43	0.53	–
RGN2 [48]	Nat. Biotechnol.’22	**1.87**	2.01	2.88	3.35	0.80	0.20	–
trRosettaX-single [49]	Nat Comput Sci’22	2.43	2.72	**2.50**	2.78	**0.88**	0.17	–
ESMFold [15]	Science’23	*1.63*	**1.24**	*2.38*	**1.62**	*0.89*	**0.15**	–
FreeProtMap	Ours	2.30	*1.20*	3.39	*2.25*	0.86	*0.13*	2.49

$MAE_{d}$ and *RMSE* units are Å. $R^2$ does not have a unit

#### Comparison with ESMFold on computational complexity

Since ESMFold achieves suboptimal performance on most datasets in the comparative experiments, we will further compare ESMFold with FreeProMap in terms of computational complexity.

We report the average time and max space cost of FreeProtMap and ESMFold on a local server in Table 2. FreeProtMap generates a distance map in 0.0295 s on average, with an input protein sequence length of 376.6 AA. Its inference time is just 3.74‰ that of ESMFold. FreeProtMap’s quick inference time is the result of its lightweight architecture and additive attention calculation. ESMFold consists of 56 blocks and requires a recycling step, but FreeProtMap only consists of 5 blocks and skips the recycling step.

**Table 2:** Complexity of proposed FreeProtMap and ESMFold

	T(representation)	T(inference)	Total time consumption	S(inference)
ESMFold	*	*	10.93	47.82G
FreeProtMap	0.0114	0.0295	0.0409	35.46G

T(representation) denotes the required time to generate and descale attention maps to form input representation. T(inference) denotes the required time for inference after feature processing. S(inference) denotes the required memory during the inference process. The unit of time is second. ‘*’ denotes the unknown time consumption. The average length of input protein sequence is 376.6 AA

### Analysis of predicted results

#### The predicted distance map reflects the structural details

**Fig. 4:**
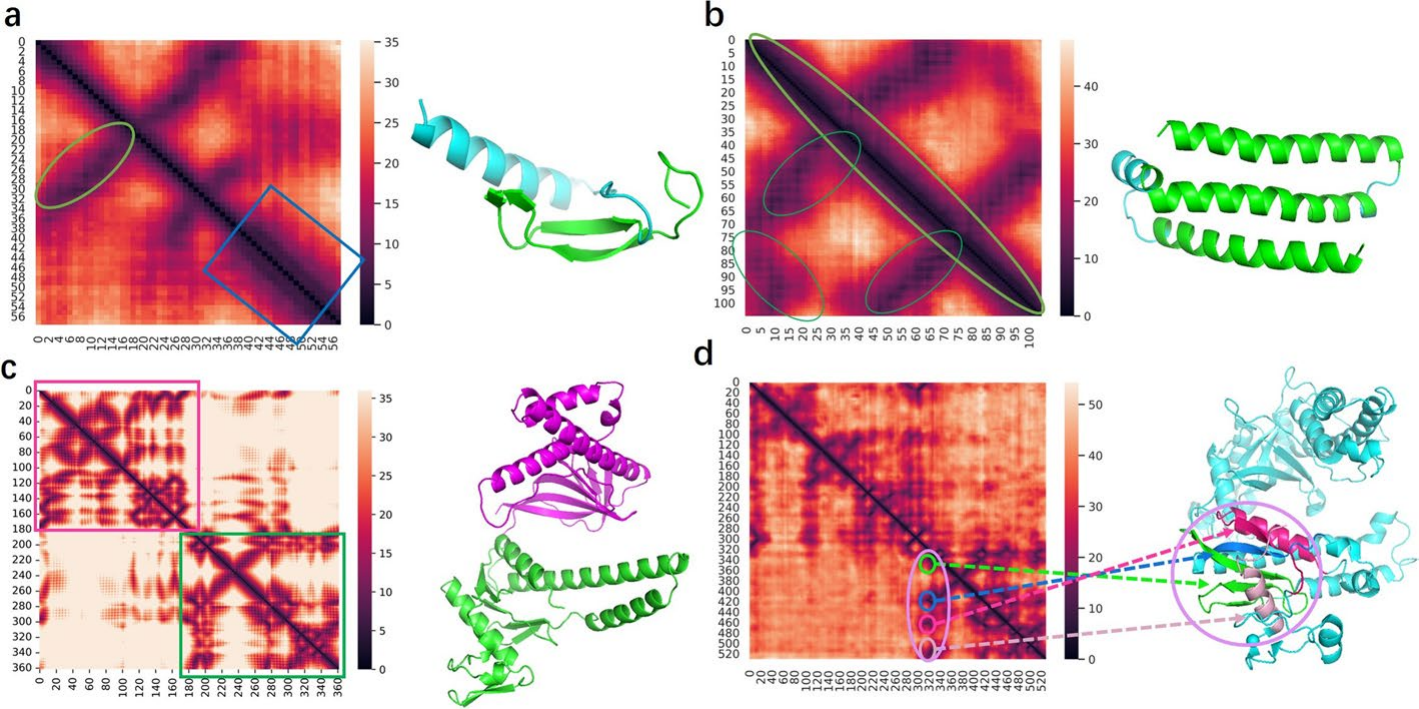
Predicted distance maps and corresponding real 3D structures. **a**, Two parallel protein fragments are in green circles and highlighted in green. An alpha helix is in a blue box and highlighted in blue. **b**, Three parallel alpha helixes are in green circles and highlighted in green. **c**, Two continuous domains are in a purple box and a green box, as well as highlighted in purple and green, respectively. **d**, A discontinuous domain is in a purple circle and highlighted in purple. It consists of four sub-domains, which are in green, blue, hot pink, and light pink circles, respectively

The distance map can clearly display the structural details of the queried protein. In the Fig. 4a, a thick diagonal line in the green box indicates an alpha helix in the protein and a line segment in the blue circle perpendicular to the diagonal line indicates a parallel structure in the protein.

In Fig. 4b, three segments, which are perpendicular or parallel to the diagonals, indicate three protein fragments are parallel to each other. The entire diagonal line in this figure is bold, indicating that the protein is composed of alpha helix.

Based on the principle of “maximum intra-domain contacts and minimum inter-domain contacts”, it can be inferred that the corresponding protein in Fig. 4c is composed of two structural domains.

As a way to demonstrate the generalization and prediction capabilities of FreeProMap on complex proteins, as well as to show more enriched information contained in distance maps, we utilized FreeProtMap to predict the distance map of cas proteins and present the results in Fig. 4d. Cas proteins are used for gene editing, which contains multiple consecutive or non-consecutive domains. When multiple line segments appear in vertical or horizontal directions in the distance map, it indicates that the relevant protein fragments are near together and may form a discontinuous domain.

To sum up, the predicted distance maps effectively contain structural information.

#### Analysis of error prediction

**Fig. 5:**
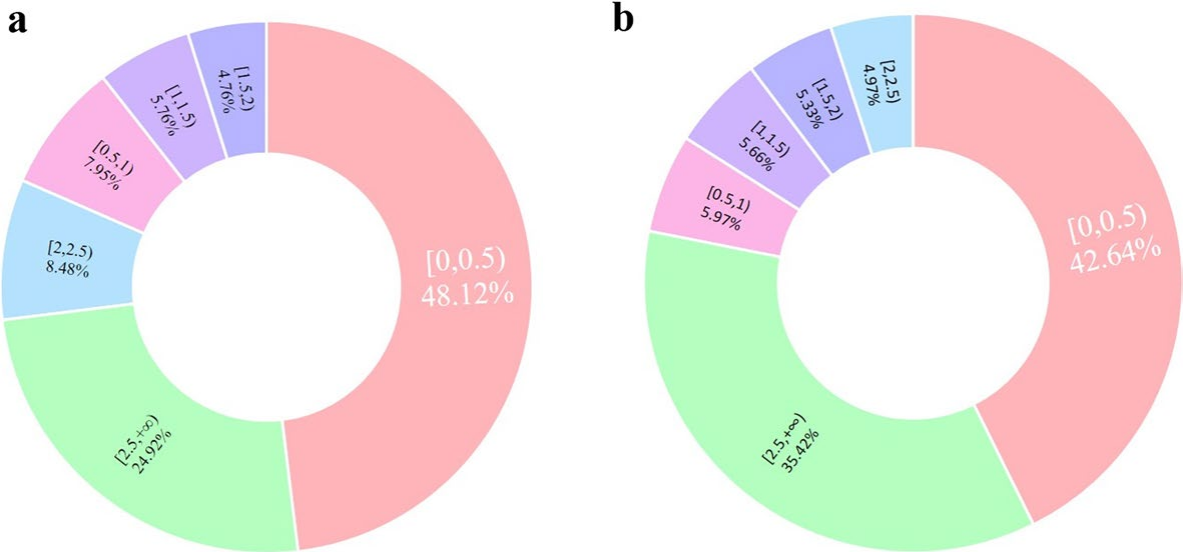
**a** The distribution of mean absolute error (MAE) between real error and predicted error. **b** The distribution of the predicted error. *Note*. The adopted statistics for error are MAE. All adopted units are Å

We report the distribution of MAE (Mean Absolute Error) between real error and predicted error (Fig. 5a), as well as the distribution of the predicted error (Fig. 5b). In 61.83% of the cases, the MAE of predicted error is below 1.5 Å. In 54.27% of the cases, the predicted error(predicted MAE) is below 1.5 Å. This suggests that FreeProtMap has some but not strong enough ability to pedicte error on predicted residue–residue distance.

### Analysis of the model

#### The evolution of feature maps

**Fig. 6:**
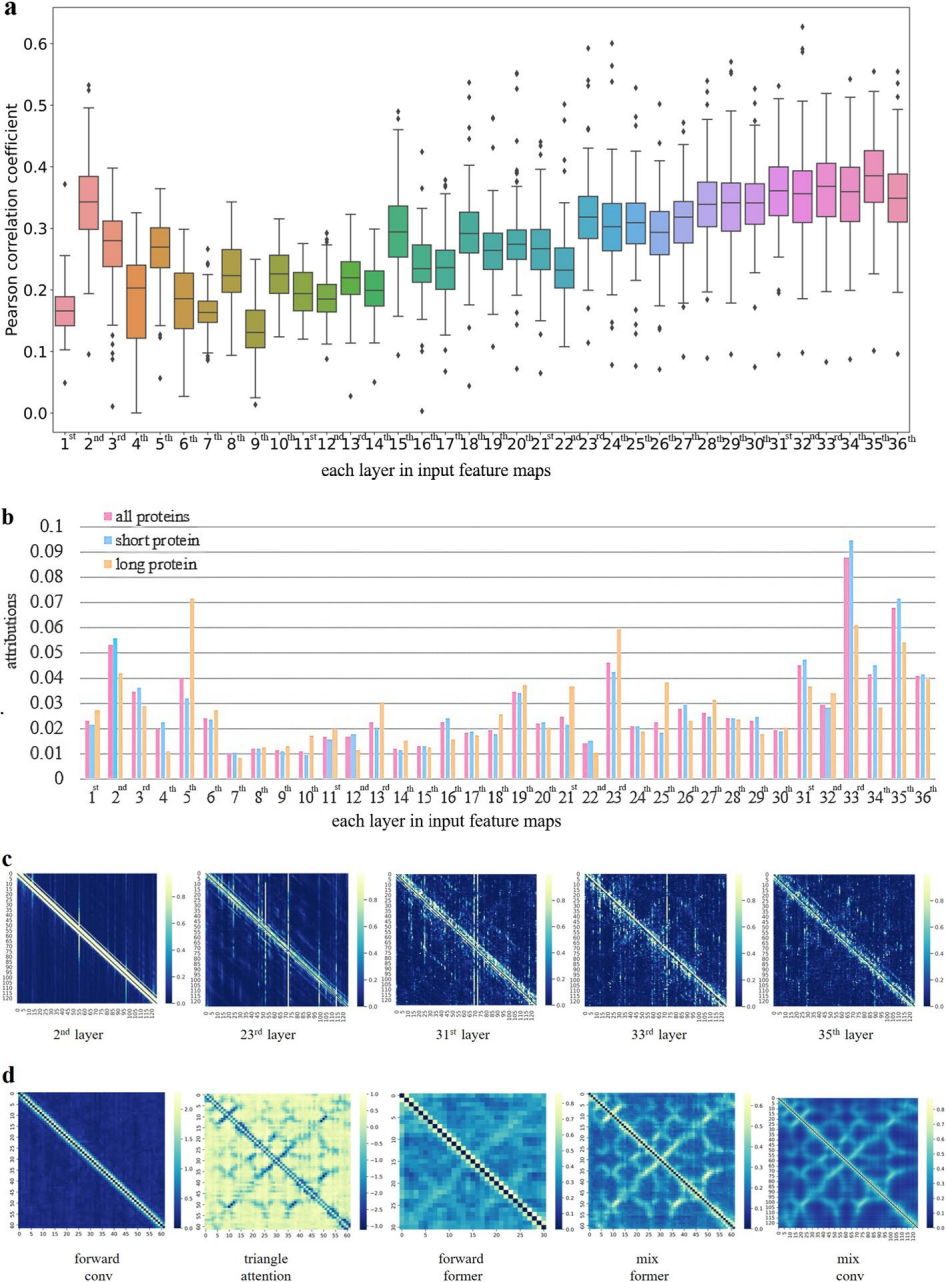
Analysis on input feature maps. **a**, The correlation between the distance map and input feature maps. **b**, Importance of each input feature maps on prediction outcomes in our approach. **c**, Top 5 input feature maps with the highest correlation to the distance map. **d**, Optimization process of input feature maps. *Note*:  ’short protein’ denotes the protein with length within [0,300 AA).  ’long protein’ denotes the the protein with length within [300,+$\infty$ AA)

We conduct a deeper analysis of the model by analyzing its feature maps. The Pearson correlation coefficients between the distance map and input r feature maps indicate that the correlation between the distance map and the 2nd, 23rd, 31st, 33rd, and 35th layer input feature maps is relatively high (Fig. 6a). The attribution of the output confirms their relatively high impact on the results (Fig. 6b). However, the depiction of aforesaid feature maps (Fig. 6c) indicates that there is a poor correlation between input feature maps and the distance map, which highlights the significance of developing an R-former to optimize input feature maps.

The mean feature maps produced by each layer of the R-former demonstrate a gradual optimization of feature maps (Fig. 6d).

#### Ablation experiment

**Fig. 7:**
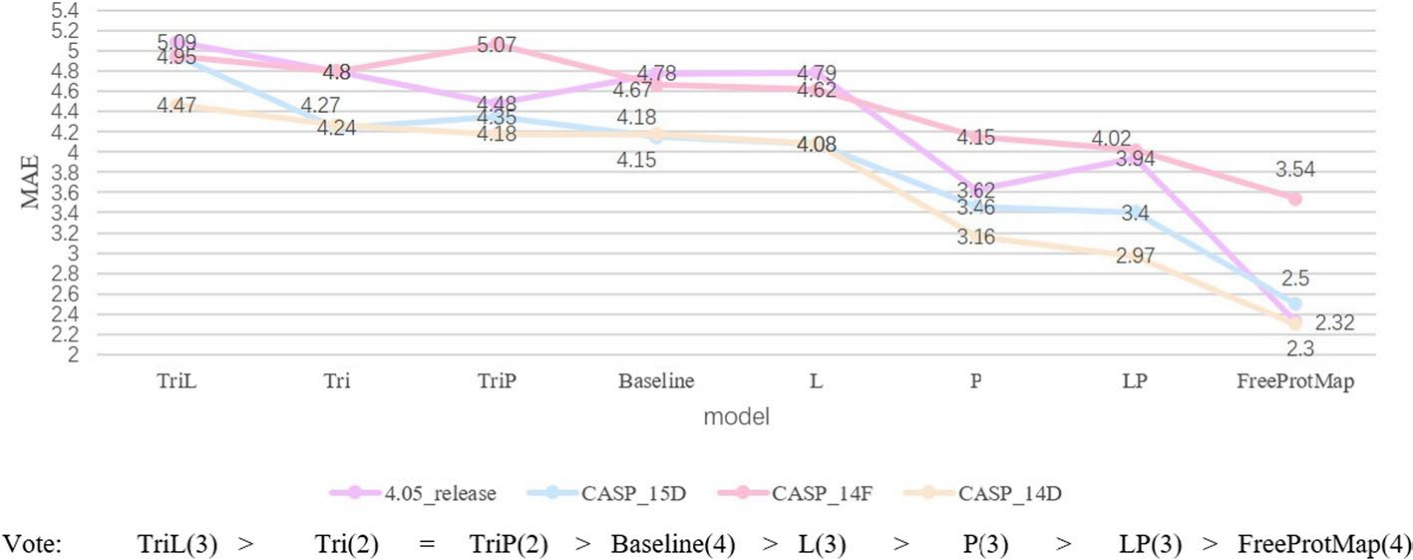
Performance of methods in ablation experiment. *Note*. The units of $MAE_{d}$ is Å

We evaluate the effectiveness of three key components: the group pooling, the former module, and the triangular attention mechanism. The ablation experimental results are summarized in Table 3 and Fig. 7.

*Baseline.* We use the FreeProtMap without the former module and triangle attention mechanism, where the input feature maps are obtained by sampling the input feature maps.

*P.* We use the FreeProtMap model without the former module and triangle attention mechanism, where the input feature maps are group-pooled attention maps.

*Tri.* We use the FreeProtMap model without the former module, where the input feature maps are obtained by sampling attention maps.

*L.* We use the FreeProtMap model without the triangle attention mechanism, where the input feature maps are obtained by sampling attention maps.

*TriL.* We use the FreeProtMap model, where the input feature maps are obtained by sampling attention maps.

*TriP.* We use the FreeProtMap model without the former module, where the input feature maps are group-pooled attention maps.

*LP.* We use the FreeProtMap model without the triangle attention mechanism, where the input feature maps are group-pooled attention maps.

*FreeProtMap.* We use our proposed methods.

**Table 3:** Results of ablation experiment

Method	4.05_release dataset					
	$MAE_{d}$	$M^d_{MAE}$	*RMSE*	$M^d_{RMSE}$	$R^2$	$M^d_{R^2}$
Baseline	4.79	0.76	7.16	0.79	0.60	0.12
P	3.62	0.54	5.63	1.01	0.75	0.08
Tri	4.82	0.92	8.60	0.94	0.36	0.31
L	4.79	0.75	7.17	0.90	0.60	0.12
TriL	5.10	0.82	7.53	0.71	0.57	0.13
TriP	4.48	0.81	6.71	0.61	0.62	0.15
LP	3.72	0.56	5.78	1.05	0.73	0.09
FreeProtMap	2.32	0.55	3.63	0.87	0.88	0.06

Method	CASP_15D					
	$MAE_{d}$	$M^d_{MAE}$	*RMSE*	$M^d_{RMSE}$	$R^2$	$M^d_{R^2}$
Baseline	4.15	2.31	6.08	3.49	0.59	0.31
P	3.46	1.88	5.24	3.00	0.68	0.26
Tri	4.24	1.45	6.13	1.80	0.58	0.23
L	4.08	1.58	6.01	2.15	0.60	0.23
TriL	4.30	1.47	6.24	1.72	0.55	0.26
TriP	3.91	1.27	5.75	1.46	0.62	0.23
LP	3.40	1.45	5.35	2.23	0.68	0.21
FreeProtMap	2.50	1.26	3.83	2.11	0.80	0.18

Method	CASP_14F					
	$MAE_{d}$	$M^d_{MAE}$	*RMSE*	$M^d_{RMSE}$	$R^2$	$M^d_{R^2}$
Baseline	4.67	1.14	6.40	1.40	0.50	0.20
P	4.15	2.42	5.79	3.62	0.58	0.34
Tri	4.80	1.26	6.54	1.56	0.47	0.23
L	4.62	1.12	6.43	1.43	0.50	0.19
TriL	4.90	1.24	6.69	1.49	0.44	0.25
TriP	4.55	1.25	6.20	1.47	0.51	0.23
LP	4.02	1.21	5.73	1.58	0.59	0.20
FreeProtMap	3.54	2.19	4.95	3.31	0.68	0.28

Method	CASP_14D					
	$MAE_{d}$	$M^d_{MAE}$	*RMSE*	$M^d_{RMSE}$	$R^2$	$M^d_{R^2}$
Baseline	4.18	0.92	6.13	0.87	0.64	0.16
P	3.16	1.94	4.73	3.27	0.77	0.22
Tri	4.27	0.93	6.25	0.92	0.63	0.16
L	4.08	0.85	6.07	0.84	0.65	0.15
TriL	4.47	0.96	6.53	0.94	0.60	0.17
TriP	3.77	0.99	5.45	1.03	0.69	0.17
LP	2.97	0.82	4.65	1.15	0.78	0.12
FreeProtMap	2.30	1.20	3.39	2.25	0.86	0.13

Table 3 and Fig. 7 show that removing group pooling has the following effects on the model’s performance:

(1) The triangular attention mechanism will lead to a decline in model performance and combining the triangular attention mechanism with the former block will significantly reduce model performance. Without the implement of group pooling to filter the input feature maps, the increased complexity of the model would ultimately undermine its performance.

(2) The former block will neither enhance nor decrease the model’s performance. On the one hand, the former block’s architecture, which improves generalization ability, keeps the model from performing worse as complexity increases. On the other hand, because the input feature maps are not adequately filtered, the former block’s role is not realized, hence adding it has no influence on model performance.

The utilization of group pooling alone significantly improved the model’s performance, primarily by mitigating the challenges associated with high-dimensional sparse data. Additionally, combining it with the former block can further improve the model’s performance. The combination of the triangle attention mechanism, the former block, and the group pooling technique make the model’s performance reach the best.

### Comparison of R-former and group pooling with similar methods

#### Comparison between group pooling and similar methods

To evaluate the power of the group pooling technique in the FreeProtMap, we compare the group pooling with the conventional dimensionality reduction method such as sampling and truncation.

*FreeProtMap_T.* It is identical to FreeProtMap except it employs truncation as the dimensionality reduction method.

*FreeProtMap_S.* It is identical to FreeProtMap except it employs sampling as the dimensionality reduction method.

**Table 4:** Comparison of dimensionality reduction methods

Method	4.05_release dataset					
	$MAE_{d}$	$M^d_{MAE}$	*RMSE*	$M^d_{RMSE}$	$R^2$	$M^d_{R^2}$
FreeProtMap_C	**4.49**	0.85	**6.65**	*0.65*	**0.64**	0.14
FreeProtMap_S	5.10	**0.82**	7.53	**0.71**	0.57	**0.13**
FreeProtMap	*2.32*	*0.55*	*3.63*	0.87	*0.88*	*0.06*

Method	CASP_15D					
	$MAE_{d}$	$M^d_{MAE}$	*RMSE*	$M^d_{RMSE}$	$R^2$	$M^d_{R^2}$
FreeProtMap_C	**4.20**	**1.41**	**6.05**	*1.63*	**0.58**	**0.24**
FreeProtMap_S	4.30	1.47	6.24	**1.72**	0.55	0.26
FreeProtMap	*2.50*	*1.26*	*3.83*	2.11	*0.80*	*0.18*

Method	CASP_14F					
	$MAE_{d}$	$M^d_{MAE}$	*RMSE*	$M^d_{RMSE}$	$R^2$	$M^d_{R^2}$
FreeProtMap_C	**4.79**	*1.22*	**6.46**	*1.45*	**0.47**	*0.24*
FreeProtMap_S	4.90	**1.24**	6.69	**1.49**	0.44	** 0.25**
FreeProtMap	*3.54*	2.19	*4.95*	3.31	*0.68*	0.28

Method	CASP_14D					
	$MAE_{d}$	$M^d_{MAE}$	*RMSE*	$M^d_{RMSE}$	$R^2$	$M^d_{R^2}$
FreeProtMap_C	**4.16**	2.22	**6.04**	3.26	**0.64**	0.28
FreeProtMap_S	4.47	*0.96*	6.53	*0.94*	0.60	**0.17**
FreeProtMap	*2.30*	**1.20**	*3.39*	**2.25**	*0.86*	*0.13*

$MAE_{d}$ and *RMSE* units are Å. $R^2$ does not have a unit

Group pooling significantly outperforms the other two methods (Table 4), because the truncation only extract features in a feature subspace and although the randomly sampled features span multiple feature subspaces, they are not complete.

#### Comparison between R-former and similar methods

To evaluate the power of the R-former module in the FreeProtMap, we compare the R-former module with several conventional networks. The distance map prediction task and the semantic segmentation task both perform regression prediction for each pixel on maps, so we choose one of the most classical segmentation networks, U-Net [50], and one of the most advanced segmentation networks, UCTrans [51], as the compared models. In addition, we also choose some simple regression models to compare, such as naive single- and multi-layer CNNs.

**Fig. 8:**
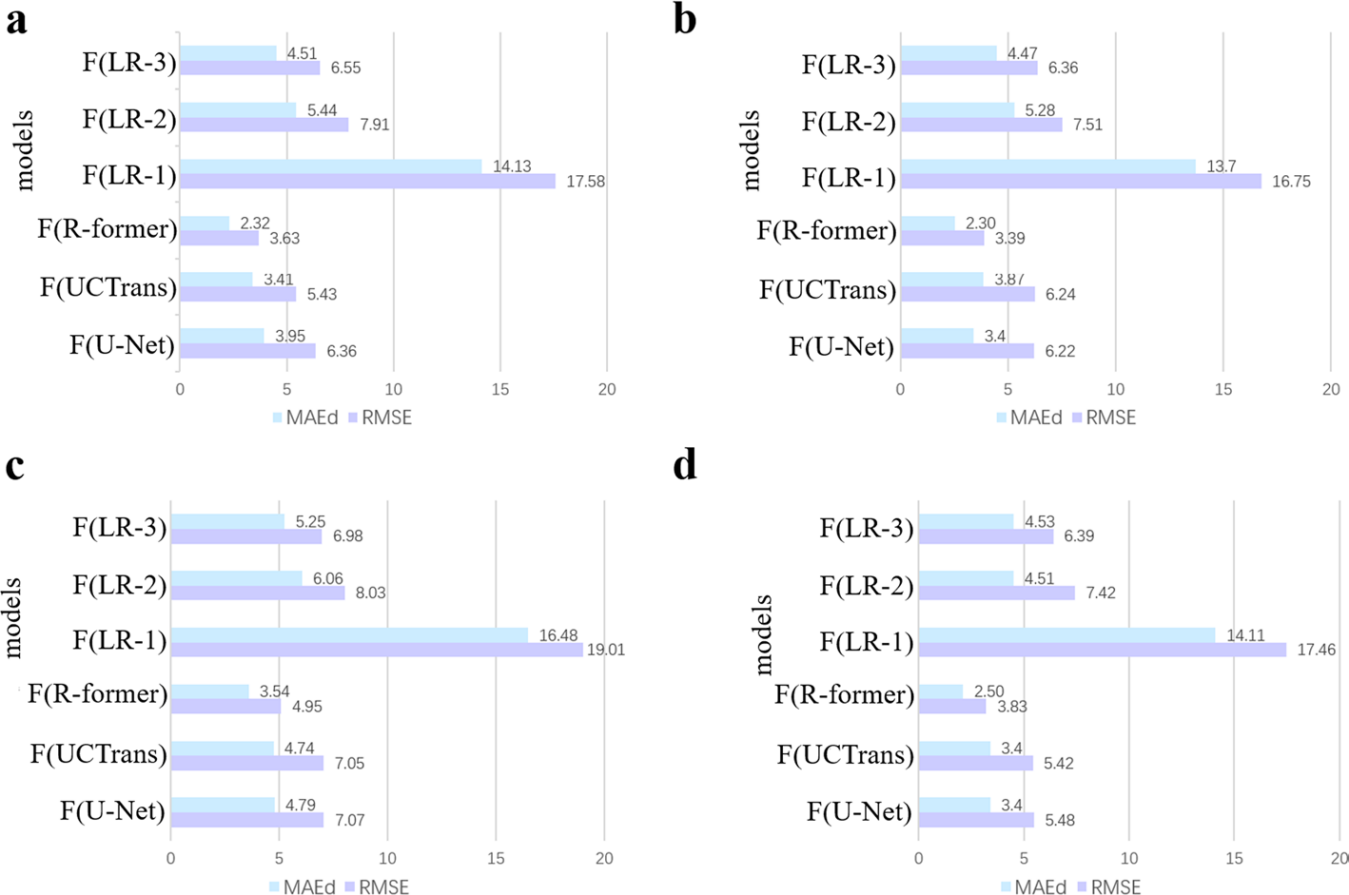
Comparison between proposed R-former and other models. **a**, Experimental result of compared models on the 4.05_release dataset. **b**, Experimental result of compared models on CASP_14D dataset. **c**, Experimental result of compared models on CASP_14F dataset. **d**, Experimental result of compared models on CASP_15D dataset. *Note*: F(U-Net), F(UCTrans) and F(LR-L) denote the proposed method, FreeProtMap, implemented by using U-Net, UCTrans and L-layer CNN as the skeleton network instead of the R-former, respectively. F(R-former) denote the proposed method, FreeProtMap, implemented by using R-former as the skeleton network. LR-L denotes L-layer CNNs with the kernel sizes of $1 \times 1$. The units of $MAE_{d}$ and *RMSE* are all Å

The results show that R-former significantly outperforms other models (Fig. 8), which indicates that our model is highly effective. More specifically, the R-former outperforms the best-compared model by 1.09 Å and 1.80 Å in $MAE_{d}$ and RMSE on the 4.05_release dataset, respectively. R-former also outperforms the best-compared model by 1.57 Å and 2.85 Å in $MAE_{d}$ and RMSE on the CASP_14D dataset, respectively. Besides, the R-former also outperforms the best-compared model by 1.20 Å and 2.10 Å in $MAE_{d}$ and *RMSE* on the CASP_14F dataset, respectively. R-former also outperforms the best-compared model by 0.90 Å and 1.59 Å in $MAE_{d}$ and *RMSE* on the CASP_15D dataset, respectively.

U-Net-like networks exceed simple models because they are more suitable to semantic-segmentation-like tasks. R-former surpasses U-Net and UCTrans for the following reasons:

1. R-former facilitates richer modeling of local information, and locality is an important characteristic of distance maps. Moreover, the former block embedded within the R-former exhibits enhanced generalization capacity [41].
2. The triangle attention module can enhance information modeling quality by imposing triangle distance inequality constraints.
3. Although UCTrans provides a richer and more efficacious way to combine information, its less generalization capacity leads to inferior results.

### Performance in contact map prediction

#### Evaluation metric

Following the standard CASP definition [40], protein residues are considered to be in contact when the inter-residue distance is less than 8.0 $\text{\AA }$ between two $C_{\beta }$ atoms ($C_{\alpha }$ for Gly). To further evaluate the performance of the proposed FreeProtMap, we convert the generated distance map into the contact map based on this threshold and compare FreeProtMap with other state-of-the-art methods for residue–residue contact prediction.

We adopt the commonly used evaluation criteria Top L/n and other criteria for classification tasks such as the Area Under the Receiver Operating Characteristic curve (AUROC), the Area Under the Precision-Recall curve (AUPR), and the F1-score(F1), because contact map prediction is a classification task.

#### Method comparison

We compare our method with existing advanced contact map prediction techniques (Tables 5, 6). The best results and the second-best results are highlighted in italic and bold, respectively. The results demonstrate that FreeProtMap significantly outperforms the best-published method on the test datasets, which verifies that our method is highly effective.

**Table 5:** The Top L/k precision of our method and compared methods

Method	Source	4.05_release dataset											
		All			Long			Medium			Short		
		L	L/2	L/5	L	L/2	L/5	L	L/2	L/5	L	L/2	L/5
ESM-1b	ICLR’21	45.39	56.35	67.12	40.27	52.08	64.81	21.07	31.10	46.46	17.35	25.82	41.34
SPOT-Contact-LM	BI’22	**75.23**	**81.65**	**86.87**	**68.07**	**76.82**	**80.89**	**68.12**	**70.17**	**78.79**	**67.69**	**68.03**	**75.07**
FreeProtMap	Ours	*77.99*	*84.33*	*88.76*	*77.21*	*80.13*	*85.00*	*73.52*	*75.00*	*81.04*	*71.79*	*72.18*	*76.01*

Method	Source	CASP_15D											
		All			Long			Medium			Short		
		L	L/2	L/5	L	L/2	L/5	L	L/2	L/5	L	L/2	L/5
ESM-1b	ICLR’21	39.33	49.47	59.67	32.11	42.11	52.93	16.52	25.27	38.64	15.05	22.52	37.48
SPOT-Contact-LM	BI’22	**72.28**	**75.05**	*80.11*	**68.27**	**74.27**	*76.48*	**67.41**	**67.43**	**72.86**	**67.12**	**67.12**	**71.58**
FreeProtMap	Ours	*73.46*	*76.07*	**78.90**	*70.54*	*74.35*	**74.64**	*72.19*	*72.19*	*75.00*	*71.05*	*71.05*	*72.89*

Method	Source	CASP_14F											
		All			Long			Medium			Short		
		L	L/2	L/5	L	L/2	L/5	L	L/2	L/5	L	L/2	L/5
ESM-1b	ICLR’21	31.36	38.55	46.83	20.48	26.39	32.54	15.10	22.55	33.33	14.42	21.48	36.51
SPOT-Contact-LM	BI’22	**68.44**	**67.23**	**66.93**	*74.16*	*77.88*	**76.81**	**61.26**	**62.00**	**63.00**	**61.39**	**61.39**	**63.65**
FreeProtMap	Ours	*71.64*	*75.52*	*78.45*	**73.85**	**76.06**	*80.94*	*64.73*	*66.62*	*72.82*	*66.15*	*66.15*	*68.05*

Method	Source	CASP_14D											
		All			Long			Medium			Short		
		L	L/2	L/5	L	L/2	L/5	L	L/2	L/5	L	L	L/5
ESM-1b	ICLR’21	66.45	77.09	83.09	47.28	62.00	75.03	36.43	50.52	71.65	36.19	51.41	70.48
SPOT-Contact-LM	BI’22	*85.34*	**88.59**	**92.46**	**65.81**	**77.85**	*90.87*	**67.63**	*79.59*	**85.34**	**77.36**	**84.87**	**85.17**
FreeProtMap	Ours	**84.99**	*90.73*	*95.42*	*71.92*	*78.79*	**87.83**	*70.89*	**77.79**	*88.51*	*78.32*	*86.95*	*90.80*

**Table 6:** Comparison of proposed approach with state-of-the-art methods for contact map prediction

Method	4.05_release dataset					
	Source	AUROC	AUPR	PRECISION	RECALL	F1
ESM-1b [37]	ICLR’21	28.22	23.77	71.34	6.95	11.49
SPOT-Contact-LM [35]	BI’22	**86.53**	**72.66**	**85.76**	**13.24**	**22.70**
FreeProtMap	Ours	*96.66*	*82.22*	*92.51*	*59.75*	*72.47*

Method	CASP_15D					
	Source	AUROC	AUPR	PRECISION	RECALL	F1
ESM-1b [37]	ICLR’21	47.30	20.19	61.01	6.16	10.75
SPOT-Contact-LM [35]	BI’22	**86.24**	**71.83**	**85.62**	**14.39**	**24.30**
FreeProtMap	Ours	*95.28*	*83.35*	*92.06*	*65.49*	*75.79*

Method	CASP_14F					
	Source	AUROC	AUPR	PRECISION	RECALL	F1
ESM-1b [37]	ICLR’21	39.66	15.13	52.01	3.65	6.47
SPOT-Contact-LM [35]	BI’22	**82.54**	**64.02**	**79.68**	**10.81**	**18.61**
FreeProtMap	Ours	*92.91*	*78.32*	*90.34*	*59.73*	*71.73*

Method	CASP_14D					
	Source	AUROC	AUPR	PRECISION	RECALL	F1
ESM-1b [37]	ICLR’21	56.05	30.10	61.42	12.24	19.83
SPOT-Contact-LM [35]	BI’22	**88.01**	**73.62**	**83.47**	**15.87**	**26.34**
FreeProtMap	Ours	*96.76*	*84.78*	*92.00*	*66.15*	*76.73*

**Fig. 9:**
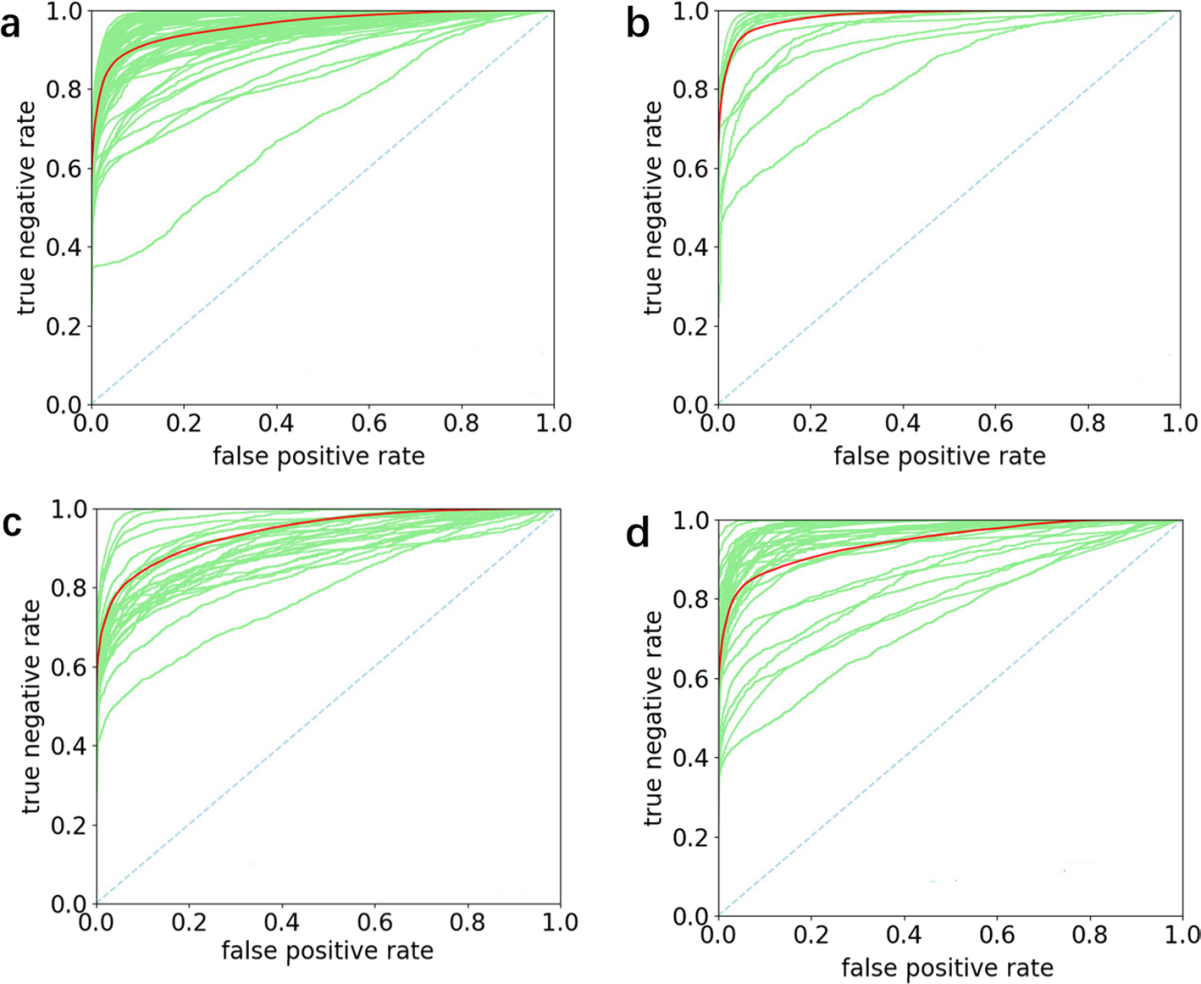
ROC curves of our method. **a**, ROC curves of FreeProtMap on the 4.05_release dataset. **b**, ROC curves of FreeProtMap on CASP_14D dataset. **c**, ROC curves of FreeProtMap on CASP_14F dataset. **d**, ROC curves of FreeProtMap on CASP_15D dataset. *Note*. ROC curves for predicted contact map of each protein are indicated in green. The mean ROC curves are indicated in red

**Fig. 10:**
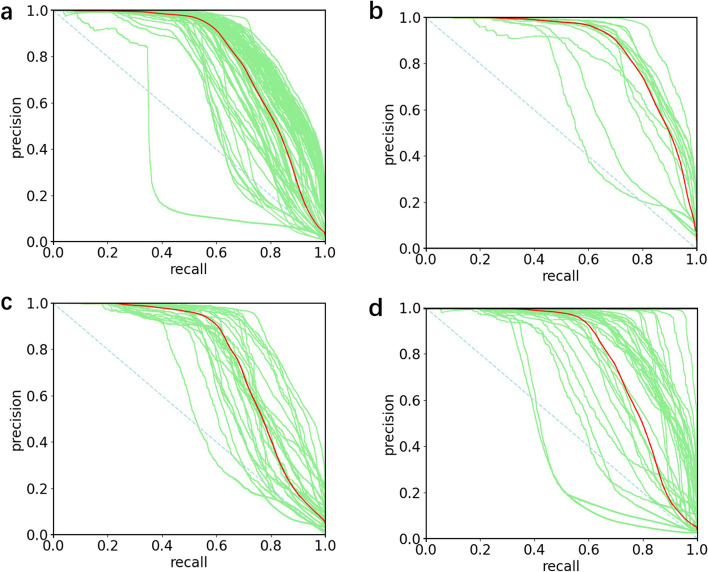
PR curves of our method. **a**, PR curves of FreeProtMap on the 4.05_release dataset. **b**, PR curves of FreeProtMap on CASP_14D dataset. **c**, PR curves of FreeProtMap on CASP_14F dataset. **d**, PR curves of FreeProtMap on CASP_15D dataset. *Note*. PR curves for predicted contact map of each protein are indicated in green. The mean PR curves are indicated in red

To comprehensively evaluate the performance of FreeProtMap, we generate individual receiver operating characteristic curve (ROC) and precision-recall curve (PR) curves for each protein, as well as mean ROC and PR curves for all proteins in each dataset (Figs. 9, 10). FreeProtMap produces favorable ROC curves for almost all tested proteins. FreeProtMap also produces favorable PR curves for 93.33%, 86.67%, 85.71% and 85.64% of the tested proteins in the 4.05_release dataset, CASP_14D dataset, CASP_14F dataset, CASP_14F dataset, respectively.

## Application prospect: remote homology protein full-scale search

The advancement of high-throughput sequencing technology has led to exponential growth in protein sequence data. Specially, hundreds of millions of proteins are discovered each year. However, due to the time-intensive process of protein structure prediction and comparison, it is difficult to conduct remote homology protein full-scale search based on their 3D structures. A common and simple solution is to construct a subset of candidate proteins with similar sequences to the reference protein and then perform remote homology protein searches within this subset by using structure prediction and comparison methods. As a result, researchers may overlook proteins that have similar structures but significantly different sequences with reference proteins, and finally it is difficult to discover new target proteins to bypass patent protection and reduce production costs.

However, we can now run a full-scale search for protein remote homology detection by using FreeProtMap for the following reasons: (1) The FreeProtMap takes $\frac{1}{400}$ the time required by ESMFold to predict distance map and exhibits higher prediction accuracy than it. (2) The distance maps possess nice properties such as rotation and translation invariance, as well as convenient comparison. We can obtain structure similarity based on the predicted distance maps.

After obtaining candidate proteins based on structure similarity by using distance maps, structure prediction models such as alphafold-2 can be used to predict the 3D structure of proteins to further analyze and select candidate proteins.

### Protein structure similarity calculation

To validate the feasibility of a protein full-scale search, we will provide specific examples and experimental results. We can obtain protein structure similarity based on distance maps by employing image comparison or distance map comparison methods. In this section, we have chosen the structure similarity index measure(SSIM) [52] to obtain protein structure similarity.

We selected three proteins with similar structures and two proteins with dissimilar structures for a reference protein as test cases, and reported the experimental results in Table 7, where the TM-score and SSIM values are calculated based on the 3D structures and distance maps, respectively. Proteins with similar structures, despite length variations, had higher TM-score and SSIM values. Conversely, proteins with dissimilar structures, although having identical lengths, have lower TM-score and SSIM values.

**Table 7:** Comparison of protein similarity calculation methods

Protein name	Length	TM-score	SSIM	T(TM-score)	T(SSIM)
1xw5_A	218	0.8906	0.7122	0.0486 s	0.0078 s
8c5d_A	209	0.9421	0.8639	0.0459 s	0.0080 s
22gs_A	211	0.9424	0.8788	0.0452 s	0.0081 s
5k6e_A	210	0.3127	0.5647	0.0978 s	0.0079 s
7qnr_A	210	0.2808	0.5638	0.0884 s	0.0089 s

The TM-score value is calculated based on the 3D structures, while the SSIM value is calculated based on the distance maps

The reference protein is 1tu7, and its sequence length is 208 AA

Assuming the average length of the query sequences is 377 amino acids (AA), it takes 46 days to generate the distance maps for one million of proteins by using an A100 GPU. It takes extra 23 days to compare the reference protein with one million of proteins based on the distance maps. If 8 A100 GPUs are employed in this task, we can scan hundreds of millions of proteins discovered each year to find structurally similar proteins in a month. However, if we perform a full-scale search by predicting and comparing 3D structures, this task will take 16 years with the same equipment.

## Conclusion

We offer FreeProtMap to make quick and accurate predictions. The proposed group pooling in FreeProtMap effectively mitigates issues arising from high-dimensional sparseness in protein representation. The proposed R-former in FreeProtMap enhances local information modeling and distance constraint relationship modeling. We will further speed up the FreeProtMap by using flash attention and expand this work to distance distribution prediction in order to assist in the molecular dynamics simulation. We also intend to enhance the performance of FreeProtMap by utilizing techniques like invariant risk minimization and error-aware loss to address sample imbalance.

### Supplementary Information

**Additional file 1: **Supplementary No. 1.

## Data Availability

All data needed to evaluate the conclusions are present in the paper. The additional data and code related to this paper can be downloaded from https://github.com/alignment-free/FreeProtMap.git.

## Footnotes

1 Helices can be identified by thickening of the diagonal line on the distance map, while parallel and antiparallel $\beta$-folds can be characterized by lines parallel or orthogonal to the diagonal line of the distance map, respectively.

2 Two or more secondary structural units are connected by a connecting polypeptide (loop) to form further a local spatial structure with a special geometric arrangement.

3 Hydrophobic, H-bond, salt bridge and so on.

4 The binary form of distance map.
