# Supplementary material for: Freeprotmap: waiting-free prediction method for protein distance map
Source: BMC Bioinformatics. 2024 May 4;25:176. doi: 10.1186/s12859-024-05771-0 (PMC11069170; doi:10.1186/s12859-024-05771-0)
Supplement: Supplementary file 1 — Additional file 1: Supplementary No. 1. [file 12859_2024_5771_MOESM1_ESM.pdf]

Appendix 1

Specifically, group-pooling is a technique used to downscale the attention maps $Attn_{input} \in R^{L \times L \times N}$ to an input representation $F_{in} \in R^{L \times L \times K}$. It is a three-step procedure:

1) The attention map $Attn_{input}$ is divided into K blocks $F_{divided} \in R^{L \times L \times N/K}$.

2) The maximum value along the channel in $F_{divided}$ is extracted to form a pooled new block $F_{pooled} \in R^{L \times L \times 1}$.

3) Subsequently, all F_{pooled} are stitched together to form an feature map $F_{in} \in R^{L \times L \times K}$. Here, L denotes the length of the input protein; N represents the number of layers in the attention map, which is 1440 in this work; K represents the number of layers in the feature map obtained from the group pooling technique, which is 36 in this work.

Appendix 2

The specific details of the triangular attention mechanism are as follows:

Algorithm 1 Triangular self-attention module

def Triangle Attention($z_{(i,j)}$):

$z_{(i,j)} = z_{(i,j)} + TriangularUpdateOutgoing(z_{(i,j)})$
$z_{(i,j)} = z_{(i,j)} + TriangularUpdateIncoming(z_{(i,j)})$
$z_{(i,j)} = z_{(i,j)} + TriangularSelfAttentionOutgoing(z_{(i,j)})$
$z_{(i,j)} = z_{(i,j)} + TriangularSelfAttentionIncoming(z_{(i,j)})$
return $z_{(i,j)}$

Algorithm 2 Detail of triangular self-attention sub-module

def TriangularUpdateOutgoing(z_{ij}, c = 128):

  $z_{ij} \leftarrow LayerNorm(z_{ij})$
  $a_{ij}, b_{ij} = sigmoid(Linear(z_{ij})) \cdot Linear(z_{ij})$
  $g_{ij} = sigmoid(Linear(z_{ij}))$
  $zij = g_{ij} \cdot Linear(LayerNorm(a_{ik} \cdot b_{jk}))$
  return z_{ij}

def TriangularUpdate**Incoming**(z_{ij}, c = 128):

  $z_{ij} \leftarrow LayerNorm(z_{ij})$
  $a_{ij}, b_{ij} = sigmoid(Linear(z_{ij})) \cdot Linear(z_{ij})$
  $g_{ij} = sigmoid(Linear(z_{ij}))$
  $z_{ij} = g_{ij} \cdot Linear(LayerNorm(\boldsymbol{a_{ki}} \cdot \boldsymbol{b_{kj}}))$
  return z_{ij}

def TriangularSelfAttentionOutgoing(z_{ij}, c = 32, N_{head} = 4):

  $z_{ij} \leftarrow LayerNorm(z_{ij})$
  $q_{ij}^h, k_{ij}^h, v_{ij}^h = LinearNoBias(z_{ij})$
  $b_{ij}^h = LinearNoBias(z_{ij})$
  $g_{ij}^h = sigmoid(Linear(z_{ij}))$
  $a_{ij}^h = softmax_k(\frac{1}{\sqrt{c}} q_{ij}^{h^T} k_{ik}^h + b_{jk}^h)$
  $o_{ij}^h = g_{ij}^h \cdot \sum_k a_{ijk}^h v_{ik}^h$
  $z_{ij} = Linear(contact_h(o_{ij}^h))$
  return z_{ij}

def TriangularSelfAttention**Incoming**(z_{ij}, c = 32, N_{head} = 4):

  $z_{ij} \leftarrow LayerNorm(z_{ij})$
  $q_{ij}^h, k_{ij}^h, v_{ij}^h = LinearNoBias(z_{ij})$
  $b_{ij}^h = LinearNoBias(z_{ij})$
  $g_{ij}^h = sigmoid(Linear(z_{ij}))$
  $a_{ij}^h = softmax_k(\frac{1}{\sqrt{c}} \boldsymbol{q_{ij}^{h^T}} \boldsymbol{k_{kj}^h} + \boldsymbol{b_{ki}^h})$
  $o_{ij}^h = g_{ij}^h \cdot \sum_k a_{ijk}^h v_{kj}^h$
  $z_{ij} = Linear(contact_h(o_{ij}^h))$
  return z_{ij}
